# Internal Carotid Artery Absence: A Case Report and Literature Review

**DOI:** 10.7759/cureus.109223

**Published:** 2026-05-19

**Authors:** Leon Hilling, Gek Shim

**Affiliations:** 1 Stroke Medicine, University Hospital of North Durham, Durham, GBR

**Keywords:** brain aneurysms, case report, congenital disease, internal carotid artery, internal carotid artery absence, internal carotid artery agenesis, literature review, neuroanatomy, vascular anomaly

## Abstract

Internal carotid artery (ICA) absence is a rare congenital vascular anomaly. The resultant collateral blood supply has been categorized into six pathways, from type A to type F. The collateral blood supply often leads to asymptomatic presentations of ICA absence. However, ICA absence is closely linked to the development of aneurysms and their severe consequences, among other signs and symptoms.

ICA absence is usually detected incidentally via computed tomography angiography (CTA) or digital subtraction angiography (DSA). There are currently no screening initiatives or treatments. In this case report, the authors will be discussing compiled evidence from various authors who have presented cases of incidentally found type A left ICA agenesis, and the surrounding literature.

## Introduction

Internal carotid artery (ICA) absence is a rare congenital vascular condition [1,2]. Less than 200 reported cases have been documented since its discovery in 1787 [3]. Patients often remain asymptomatic due to a well-established collateral blood supply [3]. The asymptomatic nature of the condition often leads to cases being found incidentally [3]. Clinical significance arises from the association between ICA absence and more severe presentations, such as aneurysms [3]. In this case report, the authors present an incidental finding of ICA absence and review the current literature on the topic.

## Case presentation

A 53-year-old male presented to the emergency department with persistent low-level vertigo and severe hypertension (blood pressure 180/100 mmHg). These symptoms were preceded by an initial acute episode of vertigo and collapse two months earlier. 

The patient’s symptoms were reviewed by the ear, nose, and throat (ENT) team. The symptoms were thought to have a peripheral aetiology, possibly secondary to vestibular neuritis. The ENT team further reported a normal ear examination and recommended magnetic resonance imaging (MRI) of the internal auditory meatus bilaterally. The scan was initially reported as showing occlusion of the left intracranial ICA, with no retrocochlear abnormalities. However, subsequent analysis revealed that the ICA was absent rather than occluded (Figure 1).

**Figure 1:**
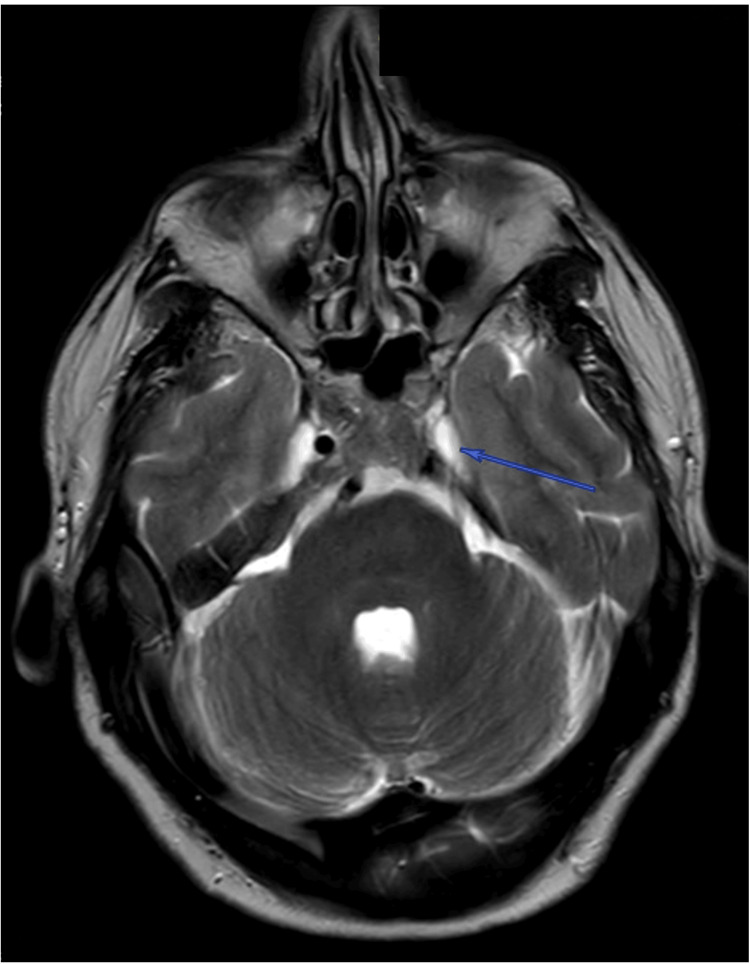
T2-weighted axial image at the level of the lacerum segment of the internal carotid artery (ICA), showing absence of the ICA flow void on the left side (blue arrow).

Discussion with the stroke team resulted in the arrangement of computed tomography angiography (CTA) of the carotid and intracranial arteries. CTA demonstrated the absence of the left ICA and the left carotid canal (Figure 2). The left common carotid artery was of smaller caliber than the right and continued directly as the left external carotid artery. The left vertebral artery was dominant compared with the right. The left middle cerebral artery (MCA) was supplied via the posterior communicating artery (PCOM), while the left anterior middle cerebral artery (AMCA) was supplied by the right anterior cerebral artery (ACA) (Figures 3-4). Additionally, there was hypoplasia of the right PCOM. Importantly, there was no filling defect, vascular malformation, or aneurysm.

**Figure 2:**
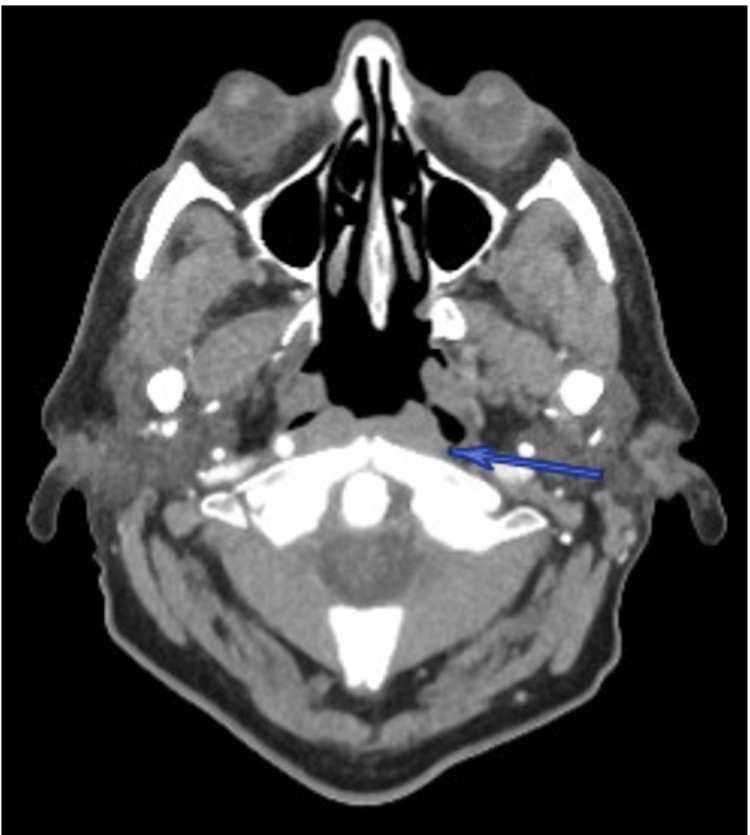
Axial computed tomography (CT) carotid angiogram image at the level of the terminal cervical internal carotid artery (ICA), showing absence of the ICA on the left side (blue arrow).

**Figure 3:**
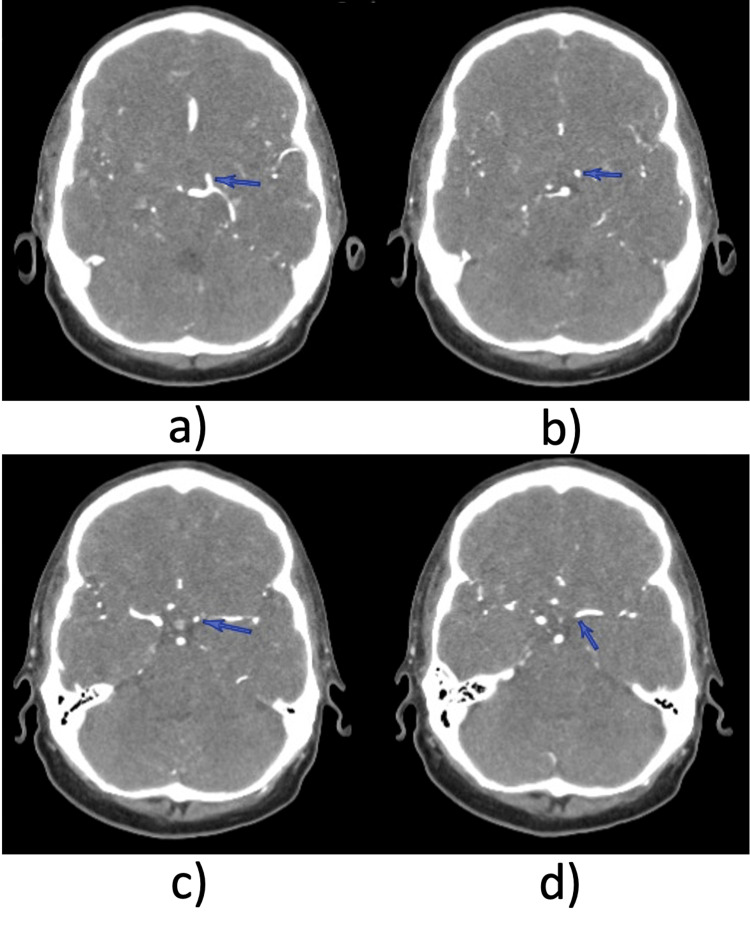
Four consecutive axial computed tomography (CT) angiogram images at the level of the Circle of Willis demonstrating that the left middle cerebral artery (MCA) is supplied by the left posterior communicating artery (PCOM). (a) Origin of the left posterior communicating artery. (b) Continuation of the left posterior communicating artery. (c) Left posterior communicating artery supplying the origin of the left MCA. (d) Left MCA. Note the absence of the supraclinoid portion of the left internal carotid artery (ICA), with preservation of the right ICA at this level.

**Figure 4:**
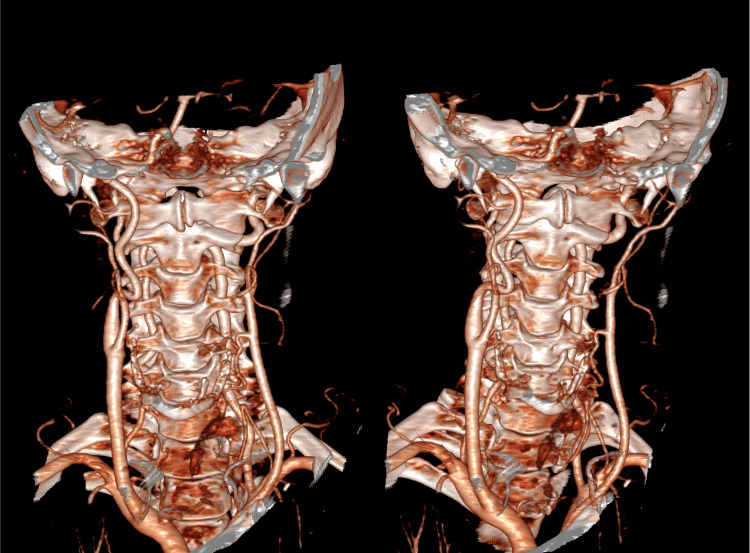
Three-dimensional reconstruction of the computed tomography (CT) angiogram demonstrating the left common carotid artery continuing as the external carotid artery, with absence of the left internal carotid artery (ICA).

Therefore, a diagnosis of congenital absence of the left ICA was made. The patient’s symptoms were thought to be caused by viral labyrinthitis, and he was treated as such. The CTA finding of congenital absence of the left ICA was treated as an incidental finding. 

## Discussion

Epidemiology

Congenital absence of the ICA is rare. Although the true prevalence is unknown, estimates suggest it occurs in fewer than 0.01% of the population [1,2]. The first reported case of ICA absence was identified postmortem by Tode in 1787 [4]. The first angiographically demonstrated case was reported by Verbiest in 1954 [5]. Since then, the literature has shown ICA absence to be more common on the left than on the right, with reported ratios of 2:1 or 3:1, respectively [6,7]. Bilateral absence of the ICA accounts for fewer than 10% of cases [6]. Zink et al. demonstrated no statistically significant sex predilection for congenital absence of the ICA, and this finding was supported by Oz et al. in a later study [7,8,9].

Nomenclature for the absence of the ICA has often been used interchangeably. Terms such as agenesis, hypoplasia, and aplasia have all been used to describe the absence of the ICA. Neurosurgeons define agenesis as the complete absence of the ICA, resulting in the absence of the carotid canal within the temporal bone. This contrasts with hypoplasia and aplasia, which describe partial formation of the ICA. In these cases, the carotid canal is present [3].

Etiology and embryology

The exact etiology of ICA agenesis is unclear. However, the resultant collateral blood flow is known to develop between the 14 mm and 24 mm stages of embryogenesis. This phase correlates with the period from completion of basilar artery formation to completion of the Circle of Willis. The stage at which ICA agenesis occurs corresponds to the type of collateral network that forms [3].

 A 1968 paper by Lie and Hage neatly outlines the resultant collateral formations following ICA agenesis or hypoplasia (Figure 5) [9]. Type A is the most common pattern and results from unilateral agenesis. The ACA on the affected side is supplied by the ACOM, while the middle cerebral artery (MCA) is supplied by the contralateral posterior cerebral artery (PCA). Type B also results from unilateral agenesis. In this pattern, the affected MCA and ACA are supplied by the ACOM, which is supplied normally by the ACA on the unaffected side. Type C results from bilateral ICA agenesis. In this case, the entire blood supply is derived from the basilar artery; therefore, both ACAs and MCAs are supplied via the posterior circulation [9].

**Figure 5:**
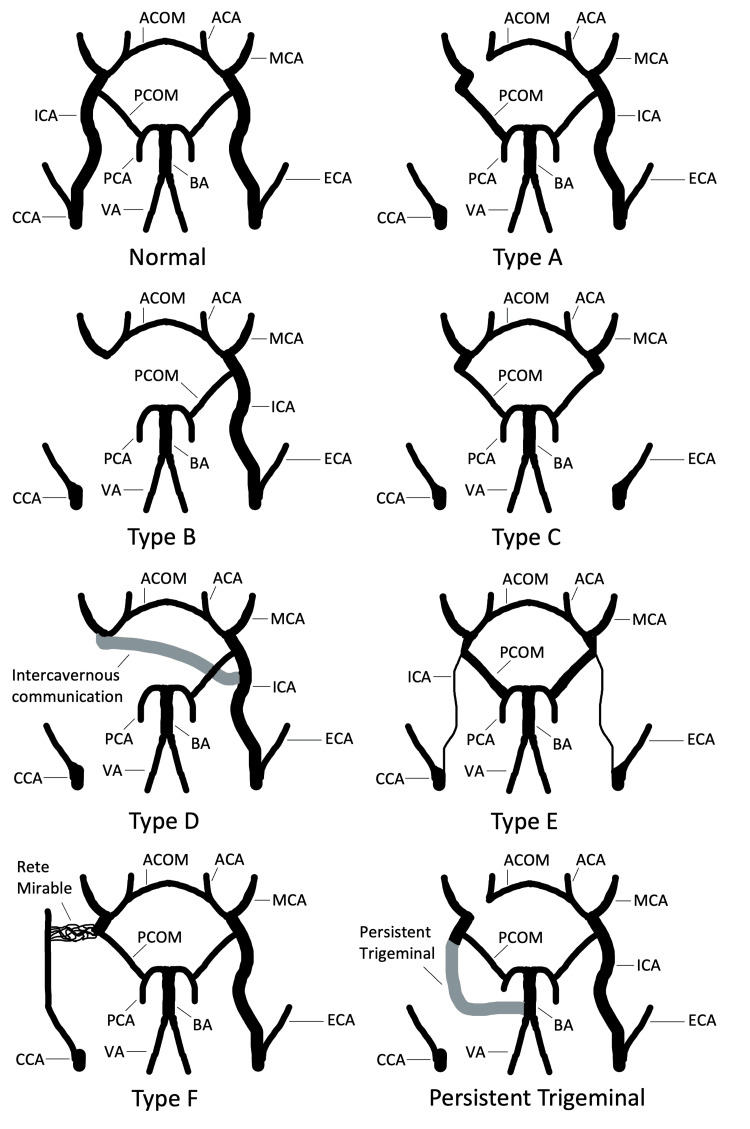
Illustrations of cerebral collateral blood flow in the absence of the ICA. Image created by Leon Hilling using Microsoft PowerPoint. ACOM, anterior communicating artery; ACA, anterior cerebral artery; MCA, middle cerebral artery; PCOM, posterior communicating artery; PCA, posterior cerebral artery; ICA, internal carotid artery; ECA, external carotid artery; CCA, common carotid artery; BA, basilar artery; VA, vertebral artery

In type D, agenesis of the cervical portion of the ICA allows a siphon from the contralateral cavernous ICA to supply the affected cavernous sinus. Type E results from bilateral hypoplasia of the ICA, in which both ICAs are of narrow caliber, whereas the PCOM is significantly wider than normal. The MCA is also of normal caliber, suggesting similarity to type C, in which the majority of the blood supply is derived from the posterior circulation. Type F results from unilateral agenesis of the ICA, in which blood supply to the ACA and MCA arises from anastomoses with the external carotid artery [9]. An additional type has also been described since the original 1968 paper. This variant was highlighted by Hattori et al. [10]. In this type, a vessel known as the persistent trigeminal artery supplies the MCA [10]. The case presented in this report would be classified as type A according to Lie et al., which is the most common classification pattern [9].

Presentation

The true incidence of congenital absence of the ICA is difficult to determine, as most cases are asymptomatic and identified only incidentally. This asymptomatic presentation is due to well-established collateral circulation replacing the function of the ICA [3]. Although formation of collateral blood supply increases the risk of aneurysm formation, Zink et al. showed that patients with ICA aplasia or hypoplasia had a 27.8% risk of developing intracranial saccular aneurysms, compared with 2%-4% in the general population. Furthermore, Zink et al. postulated that this increased risk may have an acquired etiology, given that aneurysms were more commonly observed in patients over 30 years of age than in those younger than 30 [7]. Patients with the absence of the ICA may present with manifestations of intracranial aneurysms, such as transient ischemic attacks (TIAs), nerve palsies, paresis, and subarachnoid hemorrhage [11].

Clinical presentation of ICA absence is not always related to the presence of aneurysms. Other features include complications of moyamoya disease, stroke, Horner syndrome, developmental delay, pituitary insufficiency, headaches, pulsatile tinnitus, seizures, and posterior fossa abnormalities [3,8,9]. However, it is important to stress that the vast majority of ICA absences are asymptomatic and identified only incidentally [3].

Diagnosis

As previously mentioned, ICA absence is a rare congenital vascular anomaly. Cases are usually identified incidentally via CTA and DSA [6]. Early recognition of ICA absence can be used to monitor for future aneurysm development and help prevent serious presentations [12].

Ning et al. mentioned the use of carotid Doppler ultrasound (CDUS) as a potential screening tool. CDUS was commonly used in the diagnosis of other cervical vascular diseases. Ning et al. argued that the low cost and noninvasive nature of CDUS made it an appropriate approach to screening [6].

However, given the rarity of this condition, a viable screening program is unlikely. Nevertheless, there may be scope for screening patients known to have ICA agenesis for future aneurysm development. More research is needed in this area to fully understand the costs and benefits of screening for congenital ICA absence.

Treatment

No treatment currently exists for congenital absence of the ICA. Due to the largely asymptomatic nature of ICA absence and the rarity of the condition, there is little incentive to develop treatment. However, the high prevalence of aneurysms in patients with ICA absence does provide a possible clinical incentive to screen this group of patients. Early recognition of aneurysms may allow early endovascular intervention before rupture [13]. It is the authors’ opinion that there is scope for further research into the screening and treatment of aneurysms among patients with ICA absence, particularly with regard to long-term outcomes. 

Analysis

From this case presentation and literature review, several analytical insights can be drawn. The initial misdiagnosis of ICA absence as a vascular occlusion on MRI was a predictable oversight. Without a dedicated angiographic view, Figure 1 presents diagnostic uncertainty between chronic ICA occlusion and ICA absence. Fortunately, CTA was able to demonstrate the absence of the carotid canal and, consequently, the absence of the ICA with diagnostic certainty. This case, therefore, highlights the importance of using angiographic imaging in cases of suspected occlusion or ICA nonvisualization, particularly in patients without risk factors for atherosclerotic disease.

As a result of the patient’s presentation, the unexpected finding of ICA absence has little bearing on future follow-up and treatment under current frameworks. This is despite the known increased risk of cerebrovascular morbidity associated with ICA absence [11]. The lack of consensus in the current literature regarding surveillance strategies raises the question of why long-term monitoring is not offered to these patients, given the known cerebrovascular implications [11]. In the authors’ opinion, a more proactive and risk-stratified approach should be adopted in the context of ICA absence. This may be particularly relevant in the case of this patient, who had a dominant posterior communicating artery. For an artery to become dominant, it is likely subjected to chronic hemodynamic stress and therefore at greater aneurysmal risk [6]. Thus, the added value of this case report lies not in the rarity itself, but in how vascular anomalies such as this can alter patient counseling, radiologic interpretation, risk stratification, and preemptive management of cerebrovascular risk.

## Conclusions

Congenital absence of the ICA is a rare vascular anomaly that is most often detected incidentally due to well-established collateral circulation. The case presented in this report highlights an example of type A left ICA agenesis. The condition was identified incidentally during the investigation of unrelated neurological symptoms. Although frequently asymptomatic, ICA absence is associated with an increased risk of intracranial aneurysm formation, underscoring the importance of accurate diagnosis and awareness of this condition. Recognition of ICA agenesis has important implications for imaging interpretation, clinical surveillance, and future cerebrovascular risk stratification. Further research is required to clarify the role of targeted screening and long-term monitoring in affected patients.
